# Supplementary figures and images for: Dose- and Sex-Dependent Bidirectional Relationship between Intravenous Fentanyl Self-Administration and Gut Microbiota
Source: Microorganisms. 2022 May 30;10(6):1127. doi: 10.3390/microorganisms10061127 (PMC9229572; doi:10.3390/microorganisms10061127)

# A

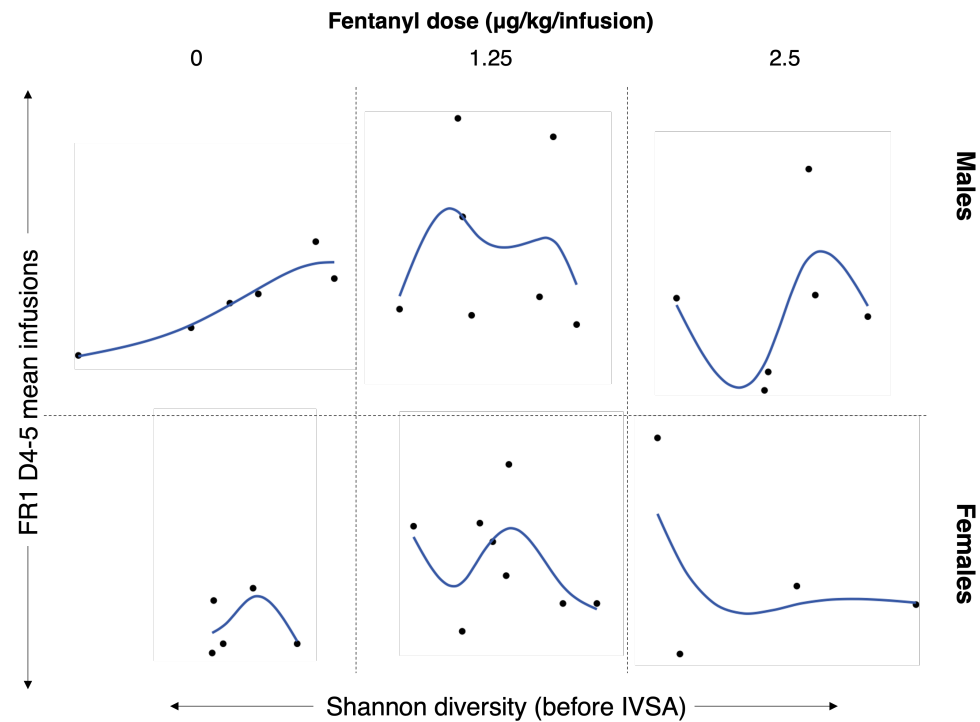

# B

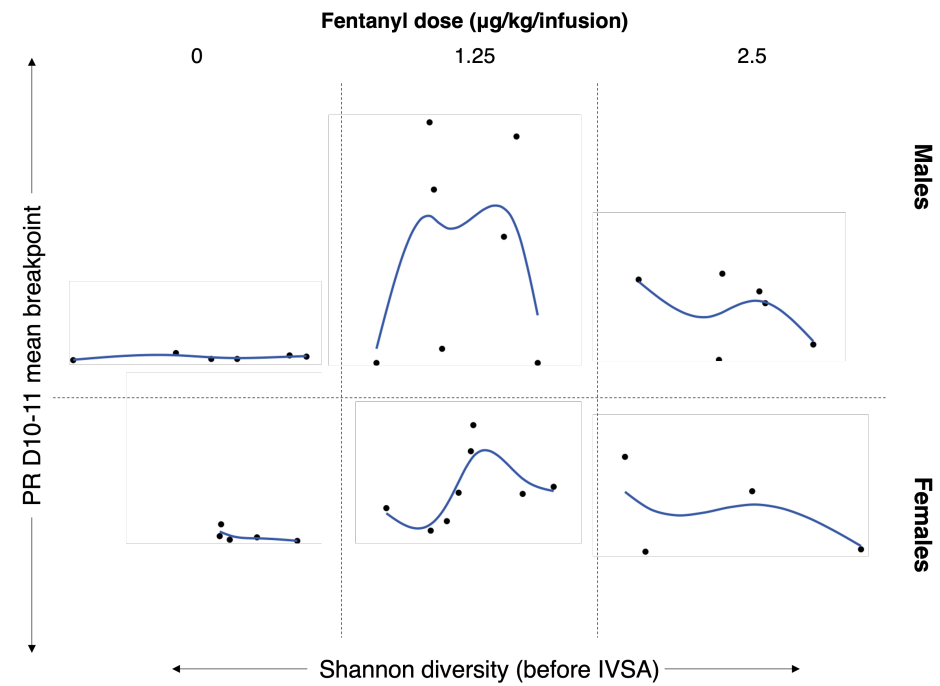

# C

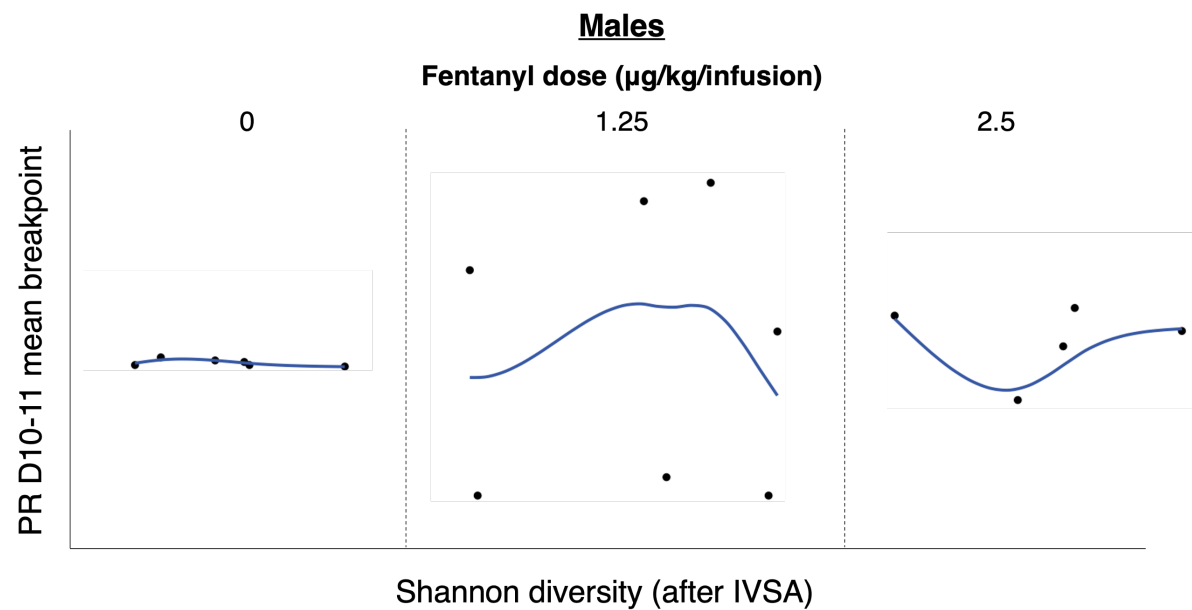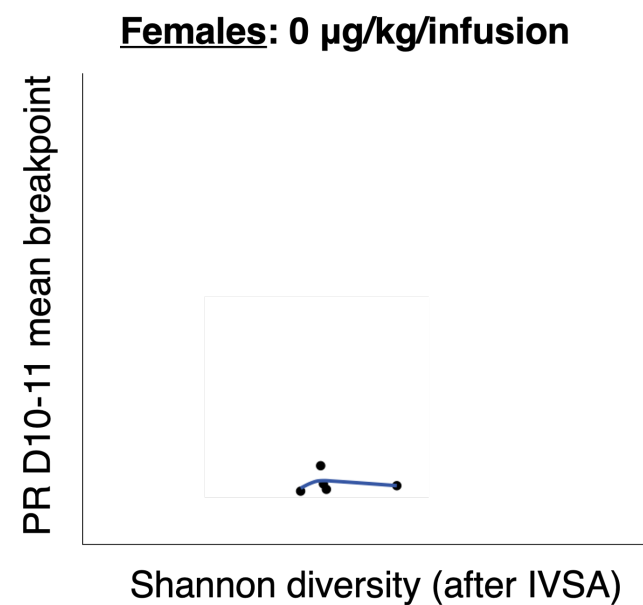

Supplement: Supplementary file 1 [file microorganisms-10-01127-s001.zip › Figure S1.pdf]

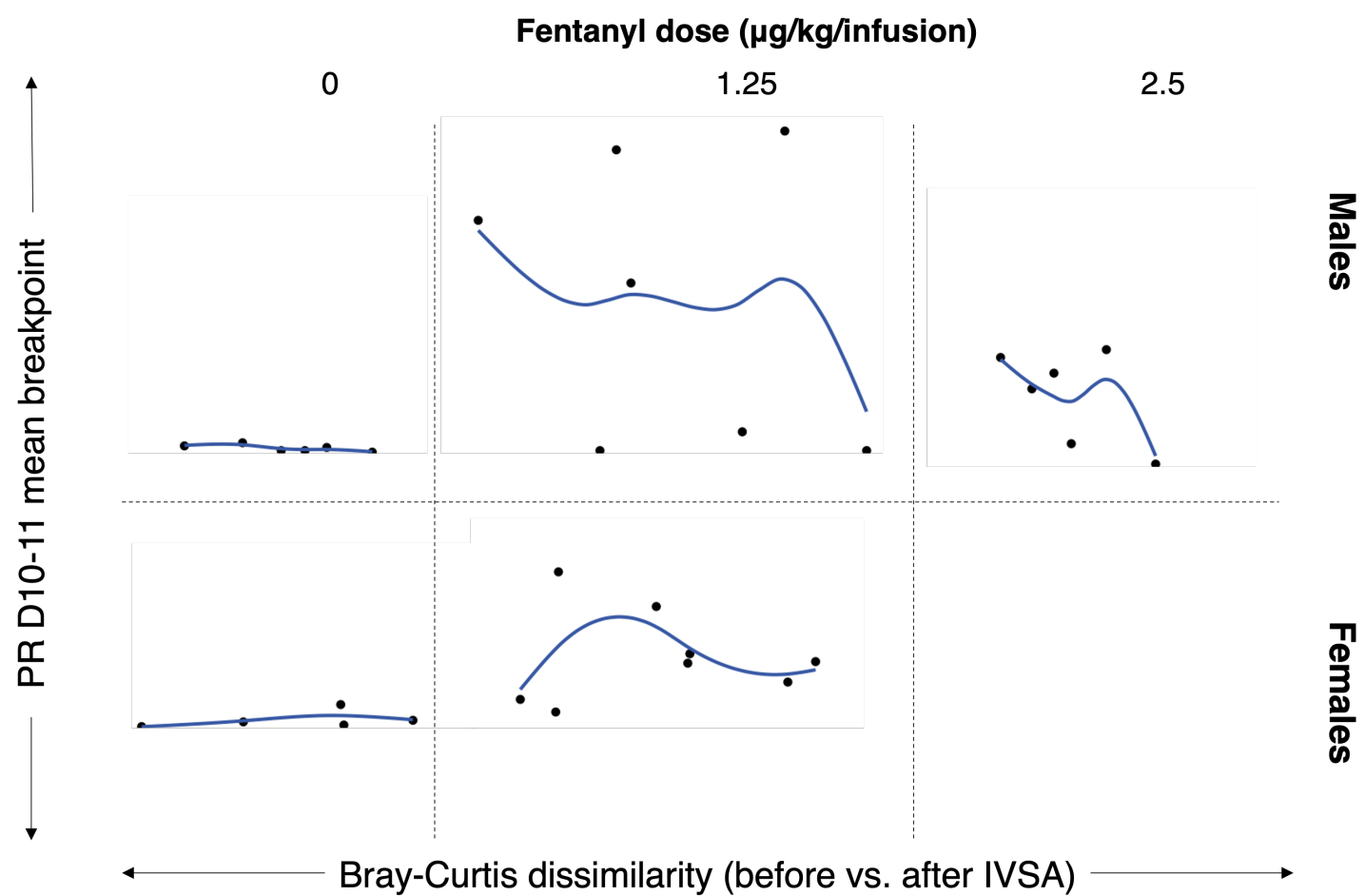

Supplement: Supplementary file 1 [file microorganisms-10-01127-s001.zip › Figure S2.pdf]
